# Emergency Department Pediatric Readiness and Short-term and Long-term Mortality Among Children Receiving Emergency Care

**DOI:** 10.1001/jamanetworkopen.2022.50941

**Published:** 2023-01-13

**Authors:** Craig D. Newgard, Amber Lin, Susan Malveau, Jennifer N. B. Cook, McKenna Smith, Nathan Kuppermann, Katherine E. Remick, Marianne Gausche-Hill, Jeremy Goldhaber-Fiebert, Randall S. Burd, Hilary A. Hewes, Apoorva Salvi, Haichang Xin, Stefanie G. Ames, Peter C. Jenkins, Jennifer Marin, Matthew Hansen, Nina E. Glass, Avery B. Nathens, K. John McConnell, Mengtao Dai, Brendan Carr, Rachel Ford, Davis Yanez, Sean R. Babcock, Benjamin Lang, N. Clay Mann

**Affiliations:** 1 Department of Emergency Medicine, Center for Policy and Research in Emergency Medicine, Oregon Health & Science University, Portland; 2 Department of Pediatrics, University of Utah School of Medicine, Salt Lake City; 3 Department of Emergency Medicine, University of California, Davis School of Medicine, Sacramento; 4 Department of Pediatrics, University of California, Davis School of Medicine, Sacramento; 5 Department of Pediatric, Dell Medical School, University of Texas at Austin, Austin; 6 Department of Surgery, Dell Medical School, University of Texas at Austin, Austin; 7 Los Angeles County Emergency Medical Services, Harbor-UCLA Medical Center, Torrance, California; 8 Centers for Health Policy, Primary Care and Outcomes Research, Department of Medicine, Stanford University School of Medicine, Palo Alto, California; 9 Division of Trauma and Burn Surgery, Department of Surgery, Children’s National Hospital, Washington, DC; 10 Department of Surgery, Indiana University School of Medicine, Indianapolis; 11 Department of Pediatrics, University of Pittsburgh School of Medicine, Pittsburgh, Pennsylvania; 12 Department of Emergency Medicine, University of Pittsburgh School of Medicine, Pittsburgh, Pennsylvania; 13 Department of Radiology, University of Pittsburgh School of Medicine, Pittsburgh, Pennsylvania; 14 Department of Surgery, Rutgers New Jersey Medical School, Newark; 15 Sunnybrook Health Sciences Centre, University of Toronto, Toronto, Ontario, Canada; 16 Center for Health Systems Effectiveness, Department of Emergency Medicine, Oregon Health & Science University, Portland; 17 Department of Emergency Medicine, Icahn School of Medicine at Mount Sinai, New York, New York; 18 Oregon Emergency Medical Services for Children Program, Oregon Health Authority, Portland; 19 Department of Anesthesia, Yale School of Medicine, New Haven, Connecticut; 20 Department of Biostatistics, Yale School of Public Health, New Haven, Connecticut

## Abstract

**Importance:**

Emergency departments (EDs) with high pediatric readiness (coordination, personnel, quality improvement, safety, policies, and equipment) are associated with lower mortality among children with critical illness and those admitted to trauma centers, but the benefit among children with more diverse clinical conditions is unknown.

**Objective:**

To evaluate the association between ED pediatric readiness, in-hospital mortality, and 1-year mortality among injured and medically ill children receiving emergency care in 11 states.

**Design, Setting, and Participants:**

This is a retrospective cohort study of children receiving emergency care at 983 EDs in 11 states from January 1, 2012, through December 31, 2017, with follow-up for a subset of children through December 31, 2018. Participants included children younger than 18 years admitted, transferred to another hospital, or dying in the ED, stratified by injury vs medical conditions. Data analysis was performed from November 1, 2021, through June 30, 2022.

**Exposure:**

ED pediatric readiness of the initial ED, measured through the weighted Pediatric Readiness Score (wPRS; range, 0-100) from the 2013 National Pediatric Readiness Project assessment.

**Main Outcomes and Measures:**

The primary outcome was in-hospital mortality, with a secondary outcome of time to death to 1 year among children in 6 states.

**Results:**

There were 796 937 children, including 90 963 (11.4%) in the injury cohort (mean [SD] age, 9.3 [5.8] years; median [IQR] age, 10 [4-15] years; 33 516 [36.8%] female; 1820 [2.0%] deaths) and 705 974 (88.6%) in the medical cohort (mean [SD] age, 5.8 [6.1] years; median [IQR] age, 3 [0-12] years; 329 829 [46.7%] female, 7688 [1.1%] deaths). Among the 983 EDs, the median (IQR) wPRS was 73 (59-87). Compared with EDs in the lowest quartile of ED readiness (quartile 1, wPRS of 0-58), initial care in a quartile 4 ED (wPRS of 88-100) was associated with 60% lower in-hospital mortality among injured children (adjusted odds ratio, 0.40; 95% CI, 0.26-0.60) and 76% lower mortality among medical children (adjusted odds ratio, 0.24; 95% CI, 0.17-0.34). Among 545 921 children followed to 1 year, the adjusted hazard ratio of death in quartile 4 EDs was 0.59 (95% CI, 0.42-0.84) for injured children and 0.34 (95% CI, 0.25-0.45) for medical children. If all EDs were in the highest quartile of pediatric readiness, an estimated 288 injury deaths (95% CI, 281-297 injury deaths) and 1154 medical deaths (95% CI, 1150-1159 medical deaths) may have been prevented.

**Conclusions and Relevance:**

These findings suggest that children with injuries and medical conditions treated in EDs with high pediatric readiness had lower mortality during hospitalization and to 1 year.

## Key Points

**Question:**

Is high emergency department (ED) pediatric readiness (6 domains of preparedness) associated with lower short-term and long-term mortality among children?

**Findings:**

In this cohort study of 796 937 children cared for in 983 EDs, there was 60% to 76% lower odds of in-hospital death associated with care in high-readiness EDs; among a subset of 545 921 children followed up beyond hospitalization, the benefit of high-readiness EDs persisted to 1 year. If all these EDs had high pediatric readiness, an estimated 1442 pediatric deaths may have been prevented.

**Meaning:**

These findings suggest that care in EDs with high pediatric readiness is associated with lower short-term and long-term mortality among children.

## Teaser

This cohort study evaluates the association between emergency department (ED) pediatric readiness, in-hospital mortality, and 1-year mortality among injured and medically ill children receiving emergency care in 11 states.

## Introduction

There are more than 30 million ED visits by children each year,^1^ representing approximately 20% of children in the US.^2^ More than 97% of EDs caring for children are nonchildren’s hospitals, accounting for 82.7% of pediatric ED visits.^3^ To address the highly variable emergency care of children,^4^ the National Pediatric Readiness Project (NPRP) was created as a national quality improvement initiative to improve the quality and consistency of care for children in US EDs.^5^ One aspect of the NPRP is increasing ED pediatric readiness, which includes care coordination, personnel and competencies, quality improvement, patient safety, policies and procedures, and availability of key equipment and supplies.^3,6^ Previous studies^3,7,8^ have shown that ED pediatric readiness varies widely among US hospitals and trauma centers, with children’s hospitals having the highest overall scores.^7^

High levels of ED pediatric readiness are associated with lower mortality among children with critical illness^9^ and those admitted to US trauma centers.^8,10^ Whether the benefits of ED pediatric readiness extend beyond these groups is unknown. Other questions include the level of ED pediatric readiness required to improve survival, whether adequately prepared EDs can save children who would die in another ED, and the potential impact of ED readiness on long-term outcomes. One study^10^ showed an association between high ED pediatric readiness and survival to 1 year among injured children admitted to trauma centers, but long-term outcomes have not been tested in other pediatric populations. Finally, the influence of hospital type and volume on the association between ED pediatric readiness and mortality remains incompletely characterized. Answers to these questions are important in determining the role of ED pediatric readiness in national health policy, hospital accreditation guidelines, and allocation of hospital resources. The objective of this study was to evaluate the association between ED pediatric readiness and in-hospital and 1-year mortality among injured and medically ill children receiving emergency care in 983 EDs in 11 states.

## Methods

### Study Design

We performed a retrospective cohort study that was reviewed and approved by institutional review boards at Oregon Health and Science University and the University of Utah School of Medicine, which waived the requirement for informed consent because the analysis was based on existing data and obtaining consent was not possible. We followed the Strengthening the Reporting of Observational Studies in Epidemiology (STROBE) reporting guidelines for cohort studies.^11^

### Study Setting

We included 983 EDs with an NPRP assessment in 11 states over a 6-year period. To be included, each ED had to care for at least 10 children requiring hospitalization over the 6 years (including children admitted to the same hospital, transferred to another hospital, or dying in the ED). The 11 states were Arizona, California, Florida, Iowa, Maryland, Minnesota, New Jersey, New York, North Carolina, Rhode Island, and Wisconsin. We selected states on the basis of broad geographic representation and availability of the necessary hospital and patient identifiers.

### Patient Population

We created a patient-level, chronological data set for consecutive children younger than 18 years receiving care in 983 EDs (records from 1 state allowed only event-level data). We identified the first ED visit for each child from January 1, 2012, through December 31, 2017, defined as the index ED visit (regardless of admission), marking time 0. The primary sample included children requiring hospitalization, transfer to another hospital, or dying in the ED during the index ED visit. For children residing in 6 states that granted approval to match state death records, we followed them for 1 year from the index ED visit (through December 31, 2018). For children transferred to another hospital, we matched available records from the second hospital to capture complete episodes of acute care. We excluded children who were treated in EDs without a matched NPRP assessment, missing hospital disposition, transferred out without a record from the second hospital, missing diagnosis codes or other key data, discharged alive from the ED, or treated in EDs that hospitalized fewer than 10 children during the 6-year study period (eFigure in Supplement 1). We divided the sample into children with injuries vs medical illnesses using hospital discharge *International Classification of Diseases, Ninth Revision, Clinical Modification (ICD-9-CM)* and *International Statistical Classification of Diseases, Tenth Revision, Clinical Modification (ICD-10-CM)* diagnosis codes to assess children with different clinical conditions and different systems of care (eg, regionalized trauma care for injured children).

### ED Pediatric Readiness

The primary exposure variable was ED pediatric readiness for the initial ED, measured using the weighted Pediatric Readiness Score (wPRS) from the 2013 NPRP assessment.^3^ We matched the NPRP assessment to the index ED record using hospital name, address, and zip code. The NPRP assessment was a national 55-question assessment based on national guidelines of US EDs providing emergency care 24 hours per day, 7 days per week, and completed by ED managers from January 1 through August 31, 2013.^3^ The wPRS is a weighted score from 0 to 100, with higher scores denoting better readiness, developed by a national expert panel using a modified Delphi process and questions with moderate to high clinical relevance for children.^12^ To create a consistent and generalizable definition for different levels of ED pediatric readiness, we calculated the quartiles of readiness (wPRS first quartile, 0-58; second quartile, 59-72; third quartile, 73-87; fourth quartile, 88-100) across all 983 EDs.

### Variables

Patient-level variables included demographics (age, sex, race, and ethnicity), complex chronic conditions,^13^ health insurance payer (a proxy of socioeconomic status), blood transfusion within 24 hours (a marker of acuity), Severity Classification System (1-5 scale, with higher numbers denoting higher clinical severity),^14^ hospital procedures, injury severity and mechanism of injury (for injured children), and interhospital transfer. We used race and ethnicity, as collected in the hospital record, to account for potential inequities in care. For hospital procedures, we used the Agency for Healthcare Research and Quality Clinical Classification System^15^ and mapped Clinical Classification System categories to standardized operative domains and blood transfusion. For injured children, we used the Abbreviated Injury Scale (AIS) score^16^ and Injury Severity Score (ISS)^17^ to measure injury severity. Because AIS and ISS are not included in administrative data, we used ICD ISS Map version 2.0 (Association for the Advancement of Automotive Medicine) to convert *ICD-9-CM* and *ICD-10-CM* diagnosis codes into standardized injury severity measures. We have previously validated ISS generated from *ICD-9-CM* diagnosis codes against hand-abstracted values.^18^

We characterized ED and hospital features using the NPRP assessment, American Hospital Association data,^19^ and patient-level data. These variables included hospital type (based on children’s hospital status, academic affiliation, trauma level, and specialty services), annual ED pediatric volume, annual pediatric ED admission volume, presence of a separate pediatric ED, and trauma center level (injured patients only).

### Outcomes

The primary outcome was in-hospital mortality, including deaths in the ED. Among children residing in 6 states, we probabilistically linked^20^ (LinkSolv version 9; Strategic Matching) state death records to generate time to death within 365 days from the index ED visit. We linked records for each state using validated linkage routines^21,22^ and variables for date of birth, home zip code, date of service, sex, race, and ethnicity. We validated the linkage results for each state using in-hospital outcomes and estimated capture rates for deaths within 1 year (eTable 1 in Supplement 1).

### Statistical Analysis

We used descriptive statistics to characterize children and hospitals by quartile of ED pediatric readiness. We performed all analyses separately for the injury and medical cohorts. Data analysis was performed from November 1, 2021, through June 30, 2022. To evaluate the association between ED pediatric readiness and in-hospital mortality, we used patient-level mixed effects logistic regression models with a random intercept to account for clustering by the initial ED,^5^ stratified by children with injury vs medical conditions (SAS statistical software version 9.4; SAS Institute). The unit of analysis was the patient. The injury model was based on a standardized risk-adjustment model for trauma,^8,23^ including age, sex, race, ethnicity, health insurance payer, comorbidities, clinical severity, blood transfusion, injury severity (ISS), mechanism of injury, transfer status, state, and year. The medical model included the same variables, except for mechanism of injury and ISS. We collapsed race to Black, White, and other (ie, American Indian, Alaska Native, Asian, Pacific Islander, or multiracial) to allow model convergence. We assessed model fit using the C statistic, influential values, and diagnostic plots. To estimate the number of additional lives that could be saved by increasing ED pediatric readiness, we used the marginal predicted probabilities of mortality by quartile of readiness to calculate the reduction in observed mortality for children cared for in lower pediatric readiness EDs (quartiles 1-3) compared with children cared for in quartile 4 EDs. We calculated 95% CIs using the bootstrap method.

We tested prespecified subgroups to assess whether children with different clinical conditions and severity of illness may be more or less sensitive to ED pediatric readiness. Among injured children, subgroups included ISS greater than or equal to 16,^17,24^ head AIS greater than or equal to 3 (serious brain injury), and Severity Classification System score greater than or equal to 4.^14^ Among children with medical conditions, subgroups included Severity Classification System score greater than or equal to 4 and different types of clinical illness (respiratory, cardiovascular, and neurologic) according to *ICD-9-CM* and *ICD-10-CM* diagnostic code groupings.^25^ We conducted stratified analyses by age group and transfer status. We tested the robustness of the results by adding hospital-level variables to the model, including hospital type, ED volume, admission volume, pediatric ED structure, and trauma center designation level (injured children).

Among children with outcomes to 1 year, we examined time to death by quartile of ED pediatric readiness using a flexible parametric model with restricted cubic splines for censored survival data^26,27^ and variance adjustment based on clustering by the initial ED^28^ implemented by the stpm2 package in Stata statistical software version 16 (StataCorp). We assessed model fit using Akaike information criterion, deviance, and Martingale residuals. As a sensitivity analysis, we repeated the models after omitting children who died in the ED and (separately) those who died within 2 days of ED presentation.

Missingness for individual variables is included in eTable 2 in Supplement 1. We used multiple imputation^29^ to handle missing values and reduce bias in the analysis. The utility and validity of multiple imputation have been shown for emergency care cohorts built from similar data sources.^22,30^ Because of differences between the injury and medical cohorts, we imputed missing data separately for the 2 cohorts using flexible chained equations, as implemented by Stata’s mi impute chained command^31,32^ and combined the results accounting for variance within and between data sets.^29,33^

## Results

Among 22 033 662 children with an ED visit during the study period (eFigure in Supplement 1), 796 937 children were hospitalized and met the inclusion criteria (admission rate, 3.6%). Of the 796 937 children, 90 963 (11.4%) were in the injury cohort and 705 974 (88.6%) were in the medical cohort. Among the 90 963 injured children (mean [SD] age, 9.3 [5.8] years; median [IQR] age, 10 [4-15] years; 33 516 [36.8%] female), 1820 (2.0%) died during their hospital stay, including 1032 in the ED (57.0% of all injury deaths). Among 705 974 children in the medical cohort (mean [SD] age, 5.8 [6.1] years; median [IQR] age, 3 [0-12] years; 329 829 [46.7%] female), 7688 (1.1%) died during their hospital stay, including 6390 in the ED (83.1% of all medical deaths). Among the 983 EDs, 592 cared for injured children and 980 cared for children with medical illness. The median (IQR) wPRS across all 983 EDs was 73 (59-87). There were 539 714 (67.7%) children treated in quartile 4 EDs, 118 917 (14.9%) in quartile 3 EDs, 72 163 (9.1%) in quartile 2 EDs, and 66 143 (8.3%) in quartile 1 EDs. Patient characteristics for each cohort by quartile of ED pediatric readiness are included in Table 1 and hospital characteristics are shown in eTable 3 in Supplement 1.

**Table 1.** Children With Injuries and Medical Conditions Presenting to EDs, by Quartile of ED Pediatric Readiness

Cohort	Children, No. (%)			
	First quartile (wPRS 0-58)	Second quartile (wPRS 59-72)	Third quartile (wPRS 73-87)	Fourth quartile (wPRS 88-100)
Injury cohort (n = 90 963 children; n = 592 EDs)				
Children, No.	6409	8458	10 950	65 146
Age group, y				
<1	418 (6.5)	461 (5.5)	831 (7.6)	6179 (9.5)
1-4	882 (13.8)	1185 (14.0)	1630 (14.9)	13 383 (20.5)
5-9	1087 (17.0)	1556 (18.4)	2089 (19.1)	14 374 (22.1)
10-12	679 (10.6)	914 (10.8)	1207 (11.0)	8415 (12.9)
13-15	1560 (24.3)	1946 (23.0)	2408 (22.0)	12 373 (19.0)
16-17	1783 (27.8)	2396 (28.3)	2785 (25.4)	10 422 (16.0)
Sex				
Female	2373 (37.0)	3012 (35.6)	3997 (36.5)	24 134 (37.1)
Male	4036 (63.0)	5446 (64.4)	6953 (63.5)	41 012 (62.9)
Race and ethnicity				
Black	694 (10.8)	1377 (16.3)	1366 (12.5)	11 212 (17.2)
Other or multiple^a^	1366 (21.3)	1954 (23.1)	2544 (23.2)	15 869 (24.4)
White	4349 (63.0)	5127 (60.6)	7040 (64.3)	38 065 (58.4)
Hispanic	1736 (27.1)	2080 (24.6)	2996 (27.4)	19 670 (30.2)
Comorbidities				
None	5922 (92.4)	8003 (94.6)	10 252 (93.6)	59 922 (92.0)
1	385 (6.0)	362 (4.3)	539 (4.9)	3873 (6.0)
≥2	102 (1.6)	93 (1.1)	159 (1.5)	1351 (2.1)
Payer				
Private	3209 (50.1)	4157 (49.2)	5118 (46.7)	28 920 (44.4)
Public	2451 (38.2)	3051 (36.1)	4388 (40.1)	28 121 (43.2)
Self-pay	552 (8.6)	707 (8.4)	900 (8.2)	3544 (5.4)
Other	198 (3.1)	543 (6.4)	544 (5.0)	4561 (7.0)
Mechanism of injury				
Firearm	260 (4.1)	263 (3.1)	278 (2.5)	1530 (2.4)
Stabbing or penetrating injury	511 (8.0)	610 (7.2)	973 (8.9)	2937 (4.5)
Assault	623 (9.7)	880 (10.4)	1081 (9.9)	6180 (9.5)
Fall	1997 (31.2)	3034 (35.9)	3990 (36.4)	23 918 (36.7)
Motor vehicle	859 (13.4)	1088 (12.9)	1179 (10.8)	8445 (13.0)
Pedestrian or bicycle	585 (9.1)	772 (9.1)	996 (9.1)	6755 (10.4)
Other	1574 (24.6)	1811 (21.4)	2453 (22.4)	15 381 (23.6)
Injury Severity Score				
0-8	4505 (70.3)	6027 (71.3)	8059 (73.6)	44 265 (68.0)
9-15	1422 (22.2)	1917 (22.7)	2240 (20.5)	15 951 (24.5)
16-24	273 (4.3)	313 (3.7)	358 (3.3)	2777 (4.3)
≥25	209 (3.3)	201 (2.4)	293 (2.7)	2153 (3.3)
Severity of illness ≥4	3327 (51.9)	4251 (50.3)	5346 (48.8)	33 338 (51.2)
Blood transfusion within 24 h	95 (1.5)	132 (1.6)	166 (1.5)	1049 (1.6%)
Major surgery^b^	584 (9.1)	610 (7.2)	831 (7.6)	5344 (8.2)
Orthopedic surgery	2486 (38.8)	3640 (43.0)	4252 (38.8)	23 215 (35.6)
Interhospital transfer	794 (12.4)	1200 (14.2)	1689 (15.4)	2114 (3.3)
In-hospital mortality	177 (2.8)	221 (2.6)	262 (2.4)	1160 (1.8)
Medical cohort (n = 705 974 children; n = 980 EDs)				
Children, No.	59 734	63 705	107 967	474 568
Age group, y				
0	18 955 (31.7)	19 226 (30.2)	36 596 (33.9)	162 140 (34.2)
1-4	10 040 (16.8)	11 910 (18.7)	20 527 (19.0)	102 577 (21.6)
5-9	7635 (12.8)	8511 (13.4)	14 373 (13.3)	72 754 (15.3)
10-12	5068 (8.5)	5384 (8.5)	9059 (8.4)	41 591 (8.8)
13-15	8983 (15.0)	9313 (14.6)	14 232 (13.2)	52 797 (11.1)
16-17	9053 (15.2)	9361 (14.7)	13 180 (12.2)	42 709 (9.0)
Sex				
Female	29 027 (48.6)	30 487 (47.9)	50 424 (46.7)	219 891 (46.3)
Male	30 707 (51.4)	33 218 (52.1)	57 543 (53.3)	254 677 (53.7)
Race and ethnicity				
Black	5890 (9.9)	10 576 (16.6)	15 297 (14.2)	89 461 (18.9)
Other or multiple^a^	12 863 (21.5)	13 435 (21.1)	31 394 (29.1)	117 032 (24.7)
White	40 981 (68.6)	39 694 (62.3)	61 276 (56.8)	268 075 (56.5)
Hispanic	18 407 (30.8)	16 981 (26.7)	33 010 (30.6)	151 077 (31.8)
Comorbidities				
None	53 751 (90.0)	56 644 (88.9)	96 795 (89.7)	389 489 (82.1)
1	4557 (7.6)	5293 (8.3)	8366 (7.8)	54 950 (11.6)
≥2	1426 (2.4)	1768 (2.8)	2806 (2.6)	30 129 (6.4)
Payer				
Private	25 268 (42.3)	25 613 (40.2)	42 514 (39.4)	209 637 (44.2)
Public	29 732 (49.8)	32 434 (50.9)	57 401 (53.2)	239 489 (50.5)
Self-pay	3617 (6.1)	4142 (6.5)	6479 (6.0)	17 770 (3.7)
Other	1117 (1.9)	1516 (2.4)	1573 (1.5)	7672 (1.6)
Most common diagnosis groups				
Respiratory	23 527 (39.4)	26 085 (41.0)	42 248 (39.1)	200 648 (42.3)
Digestive	15 921 (26.7)	17 789 (27.9)	27 746 (25.7)	138 363 (29.2)
Endocrine, nutrition, metabolic	13 521 (22.6)	15 937 (25.0)	22 244 (20.6)	130 282 (27.5)
Infection	8627 (14.4)	9259 (14.5)	15 782 (14.6)	85 216 (18.0)
Mental and behavioral	11 964 (20.0)	11 639 (18.3)	18 101 (16.8)	68 369 (14.4)
Severity of illness ≥4	31 403 (52.6)	33 756 (53.0)	54 473 (50.5)	257 942 (54.4)
Blood transfusion within 24 h	194 (0.3)	508 (0.8)	667 (0.6)	4191 (0.9)
Major surgery^b^	8866 (14.8)	9342 (14.7)	14 837 (13.7)	57 173 (12.1)
Orthopedic surgery	231 (0.4)	323 (0.5)	469 (0.4)	3154 (0.7)
Interhospital transfer	6150 (10.3)	7720 (12.1)	10 886 (10.1)	12 749 (2.7)
In-hospital mortality	1117 (1.9)	1266 (2.0)	1615 (1.5)	3690 (0.8)

Abbreviations: ED, emergency department; wPRS, weighted Pediatric Readiness Score.

^a^ Other includes American Indian, Alaska Native, Asian, and Pacific Islander.

^b^ Major surgery included brain, spine, thoracic, abdominal, pelvic, and neck procedures.

**Figure 1.**
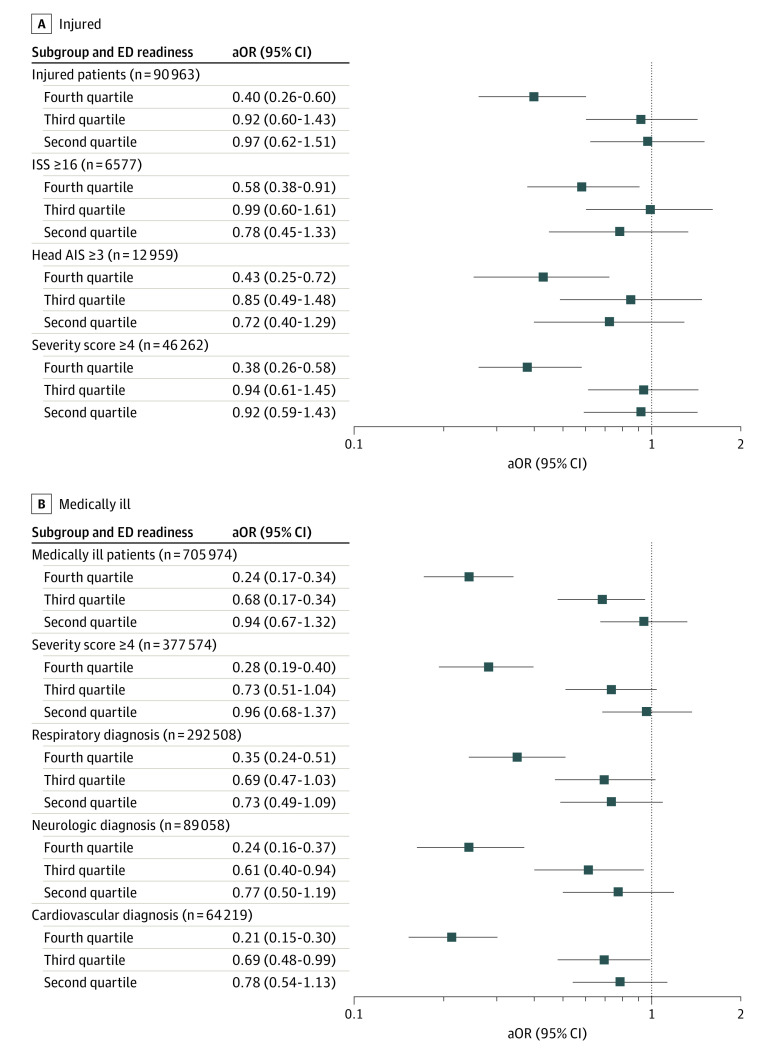
Adjusted Odds Ratios (aORs) for In-Hospital Mortality Among Children With Injuries and Medical Conditions Across Quartiles of Emergency Department (ED) Pediatric Readiness, Including Subgroups We measured ED pediatric readiness using the weighted Pediatric Readiness Score (wPRS). The reference group for all analyses was the first quartile of ED pediatric readiness (wPRS score of 0-58). The x-axis is in the natural logarithm (ln) scale. Results are shown for the injury cohort (A) and medical cohort (B). The Severity Classification System score ranges from 1 to 5, with scores of 4 or higher representing high clinical severity. AIS indicates Abbreviated Injury Scale; ISS, Injury Severity Score.

The adjusted odds of dying in hospital were 60% lower among children cared for in high-readiness EDs in the injury cohort (wPRS quartile 4 vs 1, adjusted odds ratio [aOR], 0.40; 95% CI, 0.26-0.60) and 76% lower in the medical cohort (aOR, 0.24; 95% CI, 0.17-0.34) (Table 2). These results were consistent across all subgroups (Figure 1). Although there was a significant dose-response association between increased ED pediatric readiness and decreased mortality (linear trend for aORs across quartiles), the association with decreased mortality was most consistent for children treated in quartile 4 EDs. We estimate that increasing all lower readiness EDs (quartiles 1-3) to high readiness (quartile 4) could have resulted in an additional 288 lives (95% CI, 281-297 lives) saved in the injury cohort and 1154 lives (95% CI, 1150-1159 lives) saved in the medical cohort. The benefit of care in high pediatric readiness EDs was evident across all age groups (eTable 4 in Supplement 1), but varied for children requiring transfer to another hospital (eTable 5 in Supplement 1). The association between high pediatric readiness EDs (quartile 4) and lower mortality remained after accounting for ED structure, ED pediatric volume, ED admission volume, hospital type, and trauma level (eTable 6 in Supplement 1), when restricted to the 589 EDs that cared for children in both cohorts (eTable 7 in Supplement 1), and when removing the 1 state with event-level data (eTable 8 in Supplement 1). Model diagnostics indicated appropriate model fit, lack of multicollinearity, and a C statistic of 0.94 for the injury cohort and 0.92 for the medical cohort.

**Table 2.** Multivariable Models of ED Pediatric Readiness and In-Hospital Mortality for Children With Injuries and Medical Conditions Presenting to EDs^a^

Variable	OR (95% CI)	
	Injured children (n = 90 963; 592 hospitals)	Medical children (n = 705 974; 980 hospitals)
ED wPRS		
First quartile (wPRS 0-58)	1 [Reference]	1 [Reference]
Second quartile (wPRS 59-72)	0.97 (0.62-1.51)	0.94 (0.67-1.32)
Third quartile (wPRS 73-87)	0.92 (0.60-1.43)	0.68 (0.48-0.95)
Fourth quartile (wPRS 88-100)	0.40 (0.26-0.60)	0.24 (0.17-0.34)
Female sex	1.03 (0.91-1.15)	0.89 (0.85-0.94)
Age group, y		
0	1 [Reference]	1 [Reference]
1-4	0.98 (0.79-1.21)	0.47 (0.44-0.51)
5-9	0.52 (0.41-0.65)	0.23 (0.21-0.25)
10-12	0.39 (0.30-0.50)	0.21 (0.19-0.23)
13-15	0.28 (0.22-0.35)	0.14 (0.13-0.16)
16-17	0.24 (0.19-0.30)	0.08 (0.07-0.09)
Race		
Black	0.98 (0.83-1.16)	1.68 (1.55-1.81)
Other	1.02 (0.88-1.19)	0.99 (0.92-1.07)
White	1 [Reference]	1 [Reference]
Hispanic ethnicity	0.88 (0.76-1.02)	0.56 (0.51-0.60)
Health insurance		
Public	1 [Reference]	1 [Reference]
Private	1.03 (0.88-1.21)	0.82 (0.76-0.87)
Self-pay	5.01 (4.14-6.07)	4.63 (4.24-5.06)
Other	0.95 (0.74-1.21)	1.39 (1.12-1.72)
Comorbid conditions		
None	1 [Reference]	1 [Reference]
1	2.40 (2.05-2.81)	1.43 (1.32-1.55)
≥2	4.08 (3.34-5.00)	3.28 (3.00-3.58)
Transfer out	0.19 (0.14-0.26)	0.02 (0.01-0.02)
Severity classification score		
1-3	1 [Reference]	1 [Reference]
4-5	4.92 (3.91-6.19)	62.77 (50.90-77.40)
Blood transfusion ≤24 h	2.18 (1.80-2.64)	3.31 (2.77-3.96)
Injury Severity Score		
0-8	1 [Reference]	NA
9-15	4.62 (3.74-5.72)	NA
16-24	13.63 (10.63-17.47)	NA
≥25	19.84 (15.66-25.13)	NA
Mechanism of injury		
Fall	1 [Reference]	NA
Gunshot wound	42.84 (31.58-58.10)	
Stabbing or penetrating injury	8.41 (5.70-12.41)	NA
Assault	3.38 (2.37-4.82)	NA
Motor vehicle	6.75 (5.13-8.89)	NA
Bicycle or pedestrian	9.16 (6.97-12.05)	NA
Other	4.82 (3.74-6.22)	NA

Abbreviations: ED, emergency department; OR, odds ratio; NA, not applicable; wPRS, weighted Pediatric Readiness Score.

^a^ The models also included fixed effects for state and year. Race was collapsed to Black, other (American Indian, Alaska Native, Asian, Pacific Islander, and multiracial), and White to allow model convergence.

**Figure 2.**
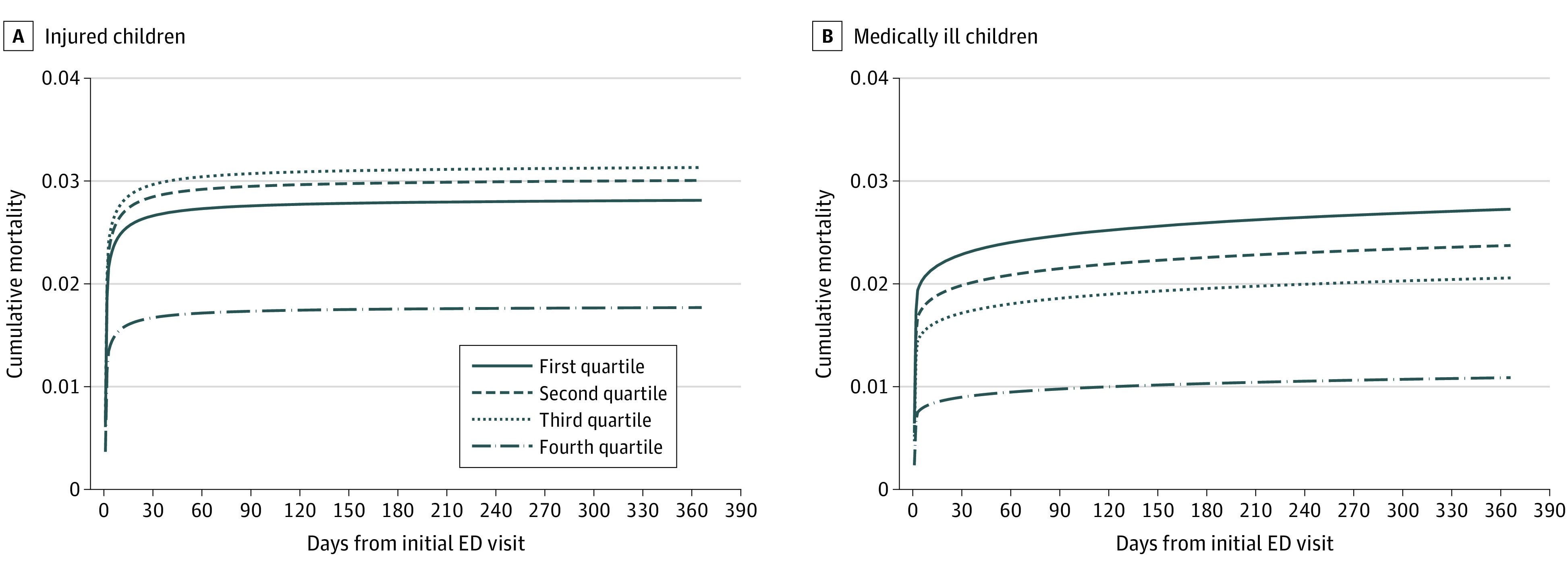
Adjusted Time to Death for Injured and Medical Children, by Emergency Department (ED) Pediatric Readiness Graphs show data for the injured cohort (A; 62 588 children) and the medical cohort (483 333 children). The adjusted hazard ratio (aHR) for death to 1 year for quartile 4 (weighted Pediatric Readiness Score [wPRS] 88-100) vs quartile 1 (wPRS 0-58) of ED pediatric readiness was 0.59 (95% CI, 0.42-0.84) for the injury cohort and was 0.34 (95% CI, 0.25-0.45) for the medical cohort. In the medical cohort, comparison of quartile 3 (wPRS 73-87) vs quartile 1 of ED pediatric readiness showed an aHR of 0.68 (95% CI, 0.51-0.92).

There were 545 921 children with outcomes to 1 year, including 62 588 (11.5%) injured children and 483 333 (88.5%) medical children. Among 1316 deaths in the injury cohort, 693 (52.7%) occurred in the ED, 477 (36.2%) as inpatients, and 146 (11.1%) following hospital discharge (2.1% cumulative 1-year mortality; median [IQR] time to death, 0 [0-2] days). Among 6635 deaths in the medical cohort, 4150 (62.5%) occurred in the ED, 759 (11.4%) as inpatients, and 1726 (26.0%) following hospital discharge (1.4% cumulative 1-year mortality; median [IQR] time to death, 0 [0-7] days). Time to death was shorter among low-readiness EDs, with 90.6% of quartile 1 injury deaths (vs 72.5% of quartile 4 deaths) and 88.5% of quartile 1 medical deaths (vs 56.8% of quartile 4 deaths) occurring within 2 days (eTable 9 in Supplement 1). After accounting for ED case mix, the risk of death to 1 year was lowest among children treated in high-readiness EDs for the injury cohort (quartile 4 vs 1 adjusted hazard ratio, 0.59; 95% CI, 0.42-0.84) and the medical cohort (adjusted hazard ratio, 0.34; 95% CI, 0.25-0.45) (Figure 2 and eTable 10 in Supplement 1). Sensitivity analyses that excluded early deaths suggested that the association between high ED readiness and lower risk of death to 1 year was primarily secondary to the prevention of early deaths (eTable 11 in Supplement 1).

## Discussion

In this cohort study, a high level of ED pediatric readiness was associated with lower short-term and long-term mortality among a heterogeneous group of hospitalized children receiving emergency care. The benefit was similar for children with different clinical conditions and severity of illness. These findings demonstrate the benefit of being adequately prepared to care for children with emergencies, regardless of hospital setting. To our knowledge, this study is the largest and most comprehensive evaluation of ED pediatric readiness to date, with major health policy implications.

Although the concept of acutely injured and ill children having lower mortality in EDs that are ready to care for them is intuitive, the evidence has been limited to children with critical illness^9^ and those admitted to trauma centers.^8,10^ The current study shows that the benefit extends to all hospitalized children cared for across a variety of settings. The mortality benefit persisted to 1 year, largely owing to the prevention of early deaths. Because the majority of children who die from acute injuries and illnesses do so early in their clinical course, EDs have the potential to change this trajectory. The mortality benefit was most consistent for EDs in the highest quartile of pediatric readiness (wPRS ≥88) and persisted after accounting for other hospital-level characteristics, suggesting a threshold effect. This finding is consistent with previous studies^8,9,10^ and provides a target for EDs seeking to raise their level of pediatric readiness.

Our results have national policy implications. National hospital accreditation organizations could consider adopting high ED pediatric readiness standards for all hospitals caring for children, with similar state-level accreditation practices. The American College of Surgeons has already introduced a requirement to assess ED pediatric readiness as part of the 2022 trauma center verification guidelines.^34^ ED pediatric readiness also could be tied to reimbursement for care. Such a value-based model would be consistent with other efforts by the Centers for Medicare & Medicaid Services and may incentivize hospitals to increase their level of ED pediatric readiness, as more than 60% of children seeking emergency care have public insurance.^1^ In addition, the level of ED pediatric readiness could be made publicly available, allowing emergency medical services, physicians, and families to select high-readiness EDs. However, this option may have unintended consequences, such as worsened care in low-readiness EDs (based on further reductions in volume, clinician skill erosion, and greater quality divides), delays in care to avoid low-readiness EDs, and further competition among hospitals. Because 30% of children live more than 30 minutes from a high-readiness ED^35^ and 27% of children transported by ambulance do not have a high-readiness ED available,^36^ the optimal solution is to promote policies and incentives to increase ED pediatric readiness among all US hospitals, including rural and frontier regions.

### Limitations

Our study has several limitations. Although the 11-state cohort included a variety of hospitals and ED practice settings, the inclusion of additional states could have changed our findings. We excluded very-low-volume EDs (ie, those admitting or transferring <10 children over 6 years), which also may have affected our findings. We used administrative state data, which can be subject to variations in coding and missing codes. However, we have validated the quality and accuracy of variables generated from similar data sources.^22,37^ It is also possible that unmeasured confounding related to the selection of EDs and ED case mix could have influenced the results. We used multiple variables to assess known and potential confounders, and our results were consistent across multiple subgroups, strata, and sensitivity analyses.

Because we used the 2013 NPRP assessment of ED pediatric readiness, it is possible that the readiness of individual EDs has changed over time. The NPRP assessment was repeated in 2021, but these data and the accompanying patient-level ED and inpatient data are not yet available. An additional study is under way to evaluate changes in ED pediatric readiness from 2013 to 2021 and the impact on pediatric mortality. Because the pediatric readiness of EDs was not independently verified, inaccuracies could have been present.

## Conclusions

In this cohort study, high ED pediatric readiness was associated with reduced in-hospital and 1-year mortality among injured and medically ill children receiving emergency care in 11 states, which appeared to be largely due to the prevention of early deaths. The findings of this study suggest that more than 1000 pediatric deaths may have been prevented in these states over 6 years by increasing the level of pediatric readiness among all EDs.

## Notes

Supplement 1. **eFigure.** Schematic of Cohort Creation **eTable 1.** Linkage Validation **eTable 2.** Missingness of Variables **eTable 3. **Hospital Characteristics by Quartile of Emergency Department (ED) Pediatric Readiness **eTable 4.** Stratified Analysis by Age Group for the Association Between ED Pediatric Readiness and In-Hospital Mortality **eTable 5.** Stratified Analysis by Transfer Status for the Association Between ED Pediatric Readiness and In-Hospital Mortality **eTable 6.** Sensitivity Analyses of Emergency Department Pediatric Readiness and In-Hospital Mortality, With the Sequential Addition of Hospital-Level Variables **eTable 7.** Multivariable Models of Emergency Department (ED) Pediatric Readiness and In-Hospital Mortality When Restricted to EDs Serving Children in the Injury and Medical Cohorts (n = 589 EDs) **eTable 8.** Multivariable Models of Emergency Department (ED) Pediatric Readiness and In-Hospital Mortality When Excluding the One State With Event-Level Data **eTable 9.** Comparison of Days-to-Death Across Quartiles of Emergency Department Pediatric Readiness Among Children Who Died Within One Year in the Injury and Medical Cohorts (n = 7,951) **eTable 10.** Time-to-Event Multivariable Spline Models **eTable 11.** Sensitivity Analyses for Time-to-Event Multivariable Spline Models That Exclude Early Deaths

Supplement 2.
Pediatric Readiness Study Group

Supplement 3.
Data Sharing Statement
