# Error in Figure 1B

**DOI:** 10.1001/jamanetworkopen.2023.1365

**Published:** 2023-02-13

In the Original Investigation titled “Emergency Department Pediatric Readiness and Short-term and Long-term Mortality Among Children Receiving Emergency Care,”^1^ published January 13, 2023, the 95% CI for the third quartile of medically ill patients in Figure 1B should have been reported as 0.48 to 0.95 instead of 0.17 to 0.34. This article has been corrected.^1^
