# Supplementary material for: Emergency Department Pediatric Readiness and Short-term and Long-term Mortality Among Children Receiving Emergency Care
Source: JAMA Netw Open. 2023 Jan 13;6(1):e2250941. doi: 10.1001/jamanetworkopen.2022.50941 (PMC9857584; doi:10.1001/jamanetworkopen.2022.50941)
Supplement: Supplement 1. — eFigure. Schematic of Cohort Creation eTable 1. Linkage Validation eTable 2. Missingness of Variables eTable 3. Hospital Characteristics by Quartile of Emergency Department (ED) Pediatric Readiness eTable 4. Stratified Analysis by Age Group for the Association Between ED Pediatric Readiness and In-Hospital Mortality eTable 5. Stratified Analysis by Transfer Status for the Association Between ED Pediatric Readiness and In-Hospital Mortality eTable 6. Sensitivity Analyses of Emergency Department Pediatric Readiness and In-Hospital Mortality, With the Sequential Addition of Hospital-Level Variables eTable 7. Multivariable Models of Emergency Department (ED) Pediatric Readiness and In-Hospital Mortality When Restricted to EDs Serving Children in the Injury and Medical Cohorts (n = 589 EDs) eTable 8. Multivariable Models of Emergency Department (ED) Pediatric Readiness and In-Hospital Mortality When Excluding the One State With Event-Level Data eTable 9. Comparison of Days-to-Death Across Quartiles of Emergency Department Pediatric Readiness Among Children Who Died Within One Year in the Injury and Medical Cohorts (n = 7,951) eTable 10. Time-to-Event Multivariable Spline Models eTable 11. Sensitivity Analyses for Time-to-Event Multivariable Spline Models That Exclude Early Deaths [file jamanetwopen-e2250941-s001.pdf]

Supplemental Online Content

Newgard CD, Lin A, Malveau S, et al; Pediatric Readiness Study Group. Emergency department pediatric readiness and short-term and long-term mortality among children receiving emergency care. *JAMA Netw Open*. 2023;6(1):e2250941. doi:10.1001/jamanetworkopen.2022.50941

eFigure. Schematic of Cohort Creation

eTable 1. Linkage Validation

eTable 2. Missingness of Variables

eTable 3. Hospital Characteristics by Quartile of Emergency Department (ED) Pediatric Readiness

eTable 4. Stratified Analysis by Age Group for the Association Between ED Pediatric Readiness and In-Hospital Mortality

eTable 5. Stratified Analysis by Transfer Status for the Association Between ED Pediatric Readiness and In-Hospital Mortality

eTable 6. Sensitivity Analyses of Emergency Department Pediatric Readiness and In-Hospital Mortality, With the Sequential Addition of Hospital-Level Variables

eTable 7. Multivariable Models of Emergency Department (ED) Pediatric Readiness and In-Hospital Mortality When Restricted to EDs Serving Children in the Injury and Medical Cohorts (n = 589 EDs)

eTable 8. Multivariable Models of Emergency Department (ED) Pediatric Readiness and In-Hospital Mortality When Excluding the One State With Event-Level Data

eTable 9. Comparison of Days-to-Death Across Quartiles of Emergency Department Pediatric Readiness Among Children Who Died Within One Year in the Injury and Medical Cohorts (n = 7,951)

eTable 10. Time-to-Event Multivariable Spline Models

eTable 11. Sensitivity Analyses for Time-to-Event Multivariable Spline Models That Exclude Early Deaths

© 2023 Newgard CD et al. *JAMA Network Open.*

This supplemental material has been provided by the authors to give readers additional information about their work.

eFigure. Schematic of Cohort Creation

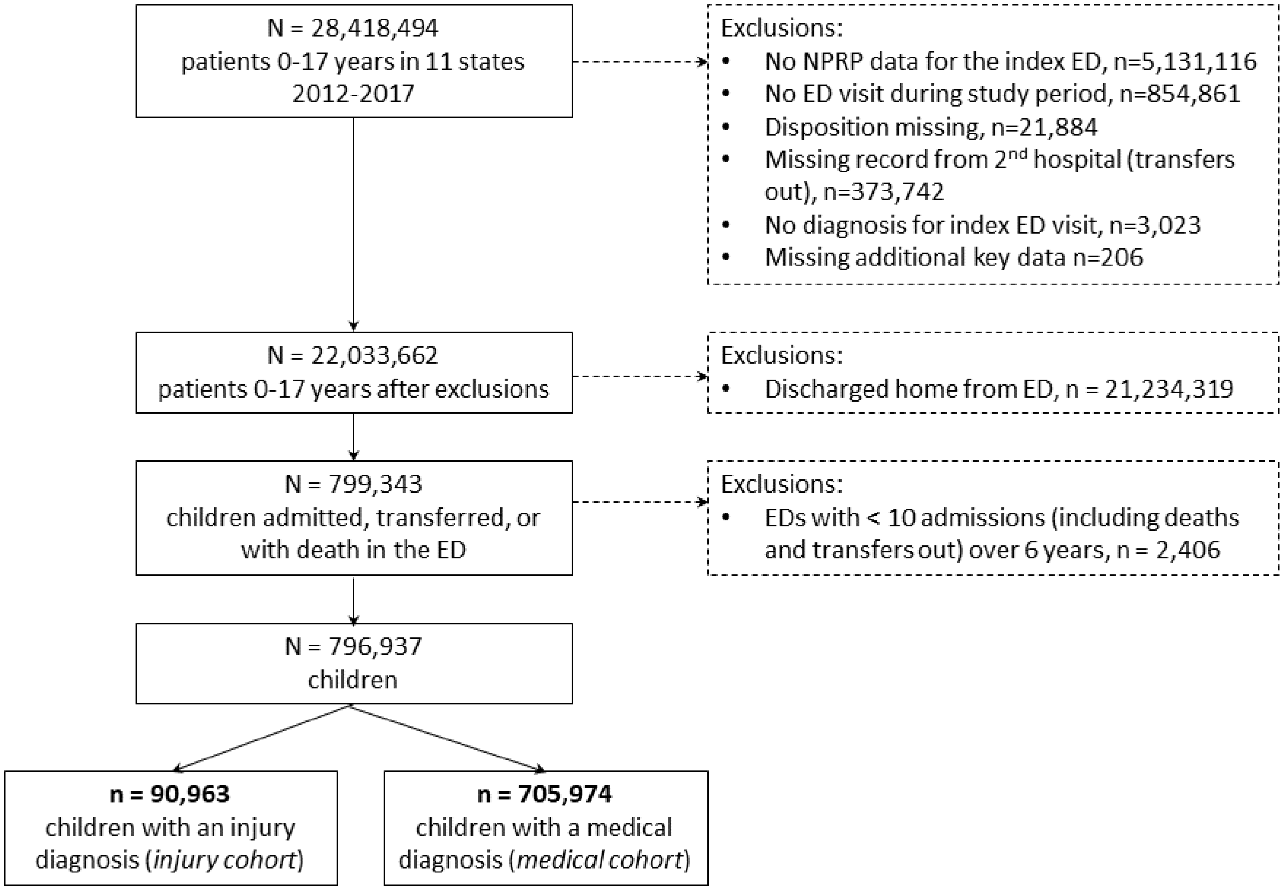

© 2023 Newgard CD et al. *JAMA Network Open.*

eTable 1. Linkage Validation

Injury cohort							
State	In-hospital deaths with LOS (state data)	Matched deaths within +/- 1 day from linked state vital statistics records	Sensitivity	In-hospital survivors with LOS (state data)	Matched deaths from vital statistics within hospital LOS for known survivors	Specificity	Estimated % of all deaths within 1 year captured
A	219	144	65.8%	7,970	1	100%	97.5%
B	377	216	57.3%	26,096	30	99.9%	86.9%
C	352	177	50.3%	13,630	10	99.9%	95.1%
D	40	30	75.0%	1,787	1	99.9%	96.1%
E	67	40	59.7%	2,646	0	100%	94.8%
F	96	61	63.5%	7,070	0	100%	95.4%
Total:	1,151	668	***58.0%***	59,199	42	***99.9%***	***92.5%***
Medical cohort							
State	In-hospital deaths with LOS (state data)	Matched deaths within +/- 1 day from linked state vital statistics records	Sensitivity	In-hospital survivors with LOS (state data)	Matched deaths from vital statistics within hospital LOS for known survivors	Specificity	Estimated % of all deaths within 1 year captured
A	747	463	62.0%	69,837	65	99.9%	85.4%
B	1,710	959	56.1%	168,948	230	99.9%	80.9%
C	1,144	666	58.2%	106,605	165	99.8%	85.7%
D	228	170	74.6%	18,201	18	99.9%	92.2%
E	490	310	63.3%	29,119	29	99.9%	91.6%
F	546	390	71.4%	72,087	50	99.9%	91.2%
Total:	4,865	2,958	***60.8%***	464,797	557	***99.9%***	***85.6%***

*LOS = length of stay. We estimated the percent of all deaths captured by calculating the total number of deaths within 365 days = in-hospital deaths + matched post-discharge deaths + additional post-discharge deaths (estimated based on state-specific match rates), then dividing the total known deaths (in-hospital and matched) by this denominator.

© 2023 Newgard CD et al. *JAMA Network Open.*

eTable 2. Missingness of Variables

	Missing (%)	
**Variable**	**Injury cohort** (n=90,963)	**Medical cohort** (n=705,974)
ED weighted Pediatric Readiness Score	0	0
Age	0	0
Sex	19 (0.02%)	45 (0.01%)
Race	9,553 (10.5%)	84,486 (12.0%)
Ethnicity	11,794 (13.0%)	100,287 (14.2%)
Health insurance payer	26,970 (29.7%)	172,158 (24.4%)
Comorbid conditions	0	0
Clinical severity score	2,218 (2.4%)	21,525 (3.1%)
Blood transfusion	0	0
Non-orthopedic surgery	0	0
Orthopedic surgery	0	0
Inter-hospital transfer	0	0
In-hospital mortality	0	0
Injury Severity Score	8,557 (9.4%)	-
Mechanism of injury	10,394 (11.4%)	-

© 2023 Newgard CD et al. *JAMA Network Open.*

eTable 3. Hospital Characteristics by Quartile of Emergency Department (ED) Pediatric Readiness

	ED weighted pediatric readiness score			
	**1st Quartile**	**2nd Quartile**	**3rd Quartile**	**4th Quartile**
	wPRS 0-58	wPRS 59-72	wPRS 73-87	wPRS 88-100
**Injury cohort (n=592 EDs)**	n=109	n=138	n=158	n=187
Trauma level				
I	5 (5%)	7 (5%)	8 (5%)	59 (32%)
II	16 (15%)	21 (15%)	28 (18%)	33 (18%)
III	22 (20%)	27 (20%)	24 (15%)	9 (5%)
IV	5 (5%)	4 (3%)	6 (4%)	1 (1%)
Non-trauma center	61 (56%)	79 (57%)	92 (58%)	85 (45%)
Pediatric trauma Level I/II	0	2 (1%)	5 (3%)	40 (21%)
Hospital type:				
Children's hospital	0	2 (1%)	1 (1%)	29 (16%)
Academic/University (non-children's hospitals)	34 (31%)	46 (33%)	65 (41%)	93 (50%)
Non-university, academic affiliated	11 (10%)	8 (6%)	7 (4%)	8 (4%)
Non-academic, Level II	6 (6%)	7 (5%)	10 (6%)	6 (3%)
Non-academic, non-Level I/II	58 (53%)	75 (54%)	75 (47%)	51 (27%)
Separate ED for children	3 (3%)	5 (4%)	25 (16%)	95 (51%)
Annual pediatric ED visits – median (IQR)	5,407 (3,253-10,333)	5,933 (3,767-10,583)	7,603 (4,481-11,109)	15,177 (8,760-21,096)
Annual pediatric admissions through the ED – median (IQR)	122 (50-357)	119 (47-341)	178 (53-602)	930 (254-2,400)
**Medical cohort (n=980 EDs)**	n=236	n=248	n=258	n=238
Trauma level				
I	6 (3%)	8 (3%)	12 (5%)	62 (26%)
II	17 (7%)	26 (10%)	31 (12%)	36 (15%)
III	46 (19%)	45 (18%)	33 (13%)	12 (5%)
IV	18 (8%)	18 (7%)	12 (5%)	4 (2%)
Non-trauma center	149 (63%)	151 (61%)	170 (66%)	124 (52%)
Pediatric trauma Level I/II	0	2 (1%)	6 (2%)	42 (18%)
Hospital type:				
Children's hospital	0	2(1%)	2 (1%)	31 (13%)
Academic/University	43 (18%)	63 (25%)	81 (31%)	108 (45%)
Non-university, academic affiliated	19 (8%)	9 (4%)	13 (5%)	10 (4%)
Non-academic, Level II	7 (3%)	11 (4%)	11 (4%)	8 (3%)
Non-academic, non-Level I/II	167 (71%)	163 (66%)	151 (59%)	81 (34%)
Separate ED for children	3 (1%)	5 (2%)	28 (11%)	101 (42%)
Annual pediatric ED visits – median (IQR)	3,251 (1,677-6,228)	4,030 (1,968-7,525)	5,230 (2,566-8,938)	12,590 (6,062-19,339)
Annual pediatric admissions through the ED – median (IQR)	38 (11-140)	47 (16-136)	72 (18-327)	558 (86-1,884)

© 2023 Newgard CD et al. *JAMA Network Open.*

eTable 4. Stratified Analysis by Age Group for the Association Between ED Pediatric Readiness and In-Hospital Mortality

	Injury cohort	Medical cohort
**0 year**	(n=7,889)	(n=236,917)
ED Pediatric Readiness Score		
1st quartile (wPRS 0-58)	referent	referent
2nd quartile (wPRS 59-72)	1.05 (0.33-3.31)	0.83 (0.53-1.30)
3rd quartile (wPRS 73-87)	0.67 (0.22-2.04)	0.53 (0.34-0.83)
4th quartile (wPRS 88-100)	0.26 (0.09-0.71)	0.16 (0.11-0.26)
**1-4 years**	(n=17,080)	(n=145,054)
ED Pediatric Readiness Score		
1st quartile (wPRS 0-58)	referent	referent
2nd quartile (wPRS 59-72)	0.75 (0.31-1.82)	1.06 (0.56-2.00)
3rd quartile (wPRS 73-87)	0.69 (0.30-1.58)	1.01 (0.54-1.87)
4th quartile (wPRS 88-100)	0.17 (0.08-0.37)	0.22 (0.12-0.41)
**5-9 years**	(n=19,106)	(n=103,273)
ED Pediatric Readiness Score		
1st quartile (wPRS 0-58)	referent	referent
2nd quartile (wPRS 59-72)	1.32 (0.49-3.53)	0.73 (0.34-1.55)
3rd quartile (wPRS 73-87)	1.24 (0.48-3.22)	0.48 (0.22-1.01)
4th quartile (wPRS 88-100)	0.41 (0.17-0.98)	0.21 (0.10-0.43)
**10-12 years**	(n=11,215)	(n=61,102)
ED Pediatric Readiness Score		
1st quartile (wPRS 0-58)	referent	referent
2nd quartile (wPRS 59-72)	0.41 (0.16-1.06)	0.65 (0.32-1.33)
3rd quartile (wPRS 73-87)	0.56 (0.23-1.34)	0.49 (0.24-0.99)
4th quartile (wPRS 88-100)	0.24 (0.11-0.49)	0.29 (0.15-0.58)
**13-15 years**	(n=18,287)	(n=85,325)
ED Pediatric Readiness Score		
1st quartile (wPRS 0-58)	referent	referent
2nd quartile (wPRS 59-72)	1.02 (0.54-1.94)	1.51 (0.82-2.80)
3rd quartile (wPRS 73-87)	0.98 (0.53-1.82)	0.97 (0.53-1.80)
4th quartile (wPRS 88-100)	0.37 (0.21-0.65)	0.53 (0.29-0.97)
**16-17 years**	(n=17,386)	(n=74,303)
ED Pediatric Readiness Score		
1st quartile (wPRS 0-58)	referent	referent
2nd quartile (wPRS 59-72)	1.29 (0.74-2.24)	1.20 (0.72-2.00)
3rd quartile (wPRS 73-87)	1.16 (0.67-2.01)	0.74 (0.44-1.25)
4th quartile (wPRS 88-100)	0.87 (0.53-1.44)	0.48 (0.30-0.79)

© 2023 Newgard CD et al. *JAMA Network Open.*

eTable 5. Stratified Analysis by Transfer Status for the Association Between ED Pediatric Readiness and In-Hospital Mortality

	**Injury cohort OR(95%CI)**	**Medical cohort OR(95%CI)**
**Non-transfer patients**		
	(n = 85,167)	(n = 668,469)
ED pediatric readiness:		
1st Quartile (least ready)	Referent	Referent
2nd Quartile	0.91 (0.54-1.52)	1.01 (0.66-1.54)
3rd Quartile	0.85 (0.51-1.40)	0.71 (0.47-1.09)
4th Quartile (most ready)	0.35 (0.22-0.55)	0.23 (0.15-0.36)
**Patients transferred to another hospital**		
	(n = 5,796)	(n = 37,505)
ED pediatric readiness:		
1st Quartile (least ready)	referent	Referent
2nd Quartile	1.67 (0.48-5.83)	0.57 (0.38-0.88)
3rd Quartile	1.49 (0.44-5.01)	0.81 (0.56-1.18)
4th Quartile (most ready)	1.09 (0.31-3.81)	0.51 (0.33-0.79)

© 2023 Newgard CD et al. *JAMA Network Open.*

eTable 6. Sensitivity Analyses of Emergency Department Pediatric Readiness and In-Hospital Mortality, With the Sequential Addition of Hospital-Level Variables

	Injury cohort	Medical cohort
	n=90,963	n=705,974
**Primary model**	OR (95%CI)	OR (95%CI)
ED Pediatric Readiness Score		
1st quartile (wPRS 0-58)	referent	referent
2nd quartile (wPRS 59-72)	0.97 (0.62-1.51)	0.94 (0.67-1.32)
3rd quartile (wPRS 73-87)	0.92 (0.60-1.43)	0.68 (0.48-0.95)
4th quartile (wPRS 88-100)	0.40 (0.26-0.60)	0.24 (0.17-0.34)
**Primary model + pediatric ED**		
ED Pediatric Readiness Score		
1st quartile (wPRS 0-58)	referent	referent
2nd quartile (wPRS 59-72)	0.97 (0.63-1.48)	0.94 (0.68-1.31)
3rd quartile (wPRS 73-87)	0.98 (0.64-1.49)	0.78 (0.56-1.09)
4th quartile (wPRS 88-100)	0.65 (0.42-0.98)	0.54 (0.37-0.77)
**Primary model + annual ED pediatric volume**		
ED Pediatric Readiness Score		
1st quartile (wPRS 0-58)	referent	referent
2nd quartile (wPRS 59-72)	0.97 (0.63-1.51)	0.98 (0.71-1.37)
3rd quartile (wPRS 73-87)	0.93 (0.60-1.42)	0.77 (0.55-1.07)
4th quartile (wPRS 88-100)	0.48 (0.32-0.73)	0.46 (0.32-0.66)
**Primary model + annual ED pediatric admission volume**		
ED Pediatric Readiness Score		
1st quartile (wPRS 0-58)	referent	referent
2nd quartile (wPRS 59-72)	0.98 (0.65-1.47)	0.95 (0.73-1.25)
3rd quartile (wPRS 73-87)	0.95 (0.64-1.40)	0.89 (0.68-1.16)
4th quartile (wPRS 88-100)	0.68 (0.46-1.00)	0.75 (0.56-1.00)
**Primary model + hospital type**		
ED Pediatric Readiness Score		
1st quartile (wPRS 0-58)	referent	referent
2nd quartile (wPRS 59-72)	1.03 (0.68-1.56)	1.00 (0.72-1.41)
3rd quartile (wPRS 73-87)	1.0 (0.67-1.50)	0.76 (0.54-1.07)
4th quartile (wPRS 88-100)	0.55 (0.37-0.82)	0.41 (0.29-0.59)
**Primary model + trauma center level**		-
ED Pediatric Readiness Score		-
1st quartile (wPRS 0-58)	referent	-
2nd quartile (wPRS 59-72)	0.97 (0.64-1.46)	-
3rd quartile (wPRS 73-87)	0.98 (0.66-1.47)	-
4th quartile (wPRS 88-100)	0.53 (0.36-0.78)	-

© 2023 Newgard CD et al. *JAMA Network Open.*

eTable 7. Multivariable Models of Emergency Department (ED) Pediatric Readiness and In-Hospital Mortality When Restricted to EDs Serving Children in the Injury and Medical Cohorts (n = 589 EDs)

	**Injured children** (n = 90,906) 589 hospitals		**Medical children** (n = 683,003) 589 hospitals
	OR (95% CI)		OR (95% CI)
ED Pediatric Readiness Score			
1st quartile (wPRS 0-58)	referent		Referent
2nd quartile (wPRS 59-72)	0.97 (0.62-1.52)		0.95 (0.63-1.45)
3rd quartile (wPRS 73-87)	0.92 (0.59-1.42)		0.72 (0.48-1.09)
4th quartile (wPRS 88-100)	0.40 (0.26-0.60)		0.27 (0.18-0.40)

© 2023 Newgard CD et al. *JAMA Network Open.*

eTable 8. Multivariable Models of Emergency Department (ED) Pediatric Readiness and In-Hospital Mortality When Excluding the One State With Event-Level Data

	**Injured children** (n = 85,108)		**Medical children** (n = 653,581)
	OR (95% CI)		OR (95% CI)
ED Pediatric Readiness Score			
1st quartile (wPRS 0-58)	referent		Referent
2nd quartile (wPRS 59-72)	1.03 (0.65-1.64)		0.95 (0.66-1.37)
3rd quartile (wPRS 73-87)	0.91 (0.58-1.42)		0.65 (0.45-0.93)
4th quartile (wPRS 88-100)	0.38 (0.25-0.58)		0.23 (0.16-0.33)

© 2023 Newgard CD et al. *JAMA Network Open.*

eTable 9. Comparison of Days-to-Death Across Quartiles of Emergency Department Pediatric Readiness Among Children Who Died Within One Year in the Injury and Medical Cohorts (n = 7,951)

ED pediatric readiness score:	n deaths	Median (IQR)	Average (SD)	% of deaths on day 0	% of deaths within 0-1 days	% of deaths within 0-2 days
Injury cohort						
1st quartile (wPRS 0-58)	117	0 (0-0)	2.0 (15.4)	75.2%	87.2%	90.6%
2nd quartile (wPRS 59-72)	172	0 (0-1)	1.8 (8.4)	71.5%	83.7%	86.6%
3rd quartile (wPRS 73-87)	204	0 (0-1)	5.8 (34.3)	67.7%	79.9%	84.3%
4th quartile (wPRS 88-100)	823	1 (0-3)	10.9 (44.1)	48.9%	65.9%	72.5%
Medical cohort						
1st quartile (wPRS 0-58)	723	0 (0-0)	7.9 (35.9)	84.4%	87.4%	88.5%
2nd quartile (wPRS 59-72)	931	0 (0-0)	11.8 (45.8)	79.4%	83.1%	84.2%
3rd quartile (wPRS 73-87)	1,137	0 (0-0)	10.5 (43.4)	77.9%	83.0%	84.9%
4th quartile (wPRS 88-100)	3,844	1 (0-26)	34.3 (72.3)	47.7%	54.5%	56.8%

© 2023 Newgard CD et al. *JAMA Network Open.*

eTable 10. Time-to-Event Multivariable Spline Models

	Injured children (n = 62,588)		Medical children (n = 483,333)
	HR (95% CI)		HR (95% CI)
ED Pediatric Readiness Score			
1st quartile (wPRS 0-58)	referent		Referent
2nd quartile (wPRS 59 to 72)	1.07 (0.73-1.56)		0.80 (0.57-1.14)
3rd quartile (wPRS 73-87)	1.13 (0.76-1.67)		0.68 (0.51-0.92)
4th quartile (wPRS 88-100)	0.59 (0.42-0.84)		0.34 (0.25-0.45)
Female	1.05 (0.92-1.19)		0.90 (0.86-0.95)
Age group			
0 years	referent		Referent
1-4 years	1.11 (0.85-1.45)		0.54 (0.49-0.60)
5-9 years	0.57 (0.46-0.72)		0.33 (0.29-0.38)
10-12 years	0.54 (0.41-0.70)		0.32 (0.27-0.37)
13-15 years	0.38 (0.29-0.50)		0.29 (0.24-0.34)
16-17 years	0.36 (0.27-0.47)		0.21 (0.18-0.25)
Race			
White	referent		Referent
Black	1.06 (0.88-1.28)		1.39 (1.25-1.54)
Other	1.05 (0.88-1.24)		0.90 (0.81-1.00)
Ethnicity - Latino	0.81 (0.67-0.98)		0.61 (0.56-0.68)
Health insurance			
Private	referent		Referent
Public	0.97 (0.79-1.19)		1.23 (1.13-1.33)
Self-pay	3.30 (2.64-4.11)		4.79 (4.23-5.43)
Other	0.85 (0.64-1.14)		1.32 (1.04-1.67)
Comorbid conditions			
none	referent		Referent
1	1.73 (1.43-2.08)		1.61 (1.43-1.82)
≥ 2	3.19 (2.34-4.34)		4.34 (3.75-5.02)
Transfer out	0.68 (0.51-0.91)		0.37 (0.31-0.44)
Severity classification score			
1-3	referent		Referent
4-5	4.77 (3.73-6.10)		14.92 (12.39-17.96)
Blood transfusion ≤ 24 hours	1.52 (1.17-1.98)		2.17 (1.75-2.69)
Injury Severity Score			-
0-8	referent		-
9-15	3.49 (2.79-4.37)		-
16-24	8.80 (6.78-11.44)		-
≥ 25	11.26 (8.65-14.66)		-
Mechanism of injury			-
Fall	referent		-
GSW	24.47 (17.62-33.98)		-
Stabbing/penetrating injury	5.65 (3.35-9.52)		-
Assault	2.83 (2.01-3.98)		-
Motor vehicle	5.26 (3.79-7.31)		-
Bicycle or pedestrian	6.75 (4.97-9.16)		-
Other	4.18 (3.07-5.68)		-

*wPRS = weighted Pediatric Readiness Score. The model included fixed effects for state and year.

© 2023 Newgard CD et al. *JAMA Network Open.*

eTable 11. Sensitivity Analyses for Time-to-Event Multivariable Spline Models That Exclude Early Deaths

	Injured children		Medical children
	OR (95% CI)		OR (95% CI)
Primary model results:	n = 62,588		n = 483,333
ED Pediatric Readiness Score:			
1st quartile (wPRS 0-58)	referent		Referent
2nd quartile (wPRS 59-72)	0.97 (0.62-1.52)		0.95 (0.63-1.45)
3rd quartile (wPRS 73-87)	0.92 (0.59-1.42)		0.72 (0.48-1.09)
4th quartile (wPRS 88-100)	0.40 (0.26-0.60)		0.27 (0.18-0.40)
Model results after excluding deaths in the ED	n =61,895 (693 of 1,316 [52.7%] deaths excluded)		n = 479,183 (n = 4,150 of 6,635 [62.5%] deaths excluded)
ED Pediatric Readiness Score:			
1st quartile (wPRS 0-58)	referent		referent
2nd quartile (wPRS 59-72)	1.26 (0.77-2.04)		1.02 (0.73-1.42)
3rd quartile (wPRS 73-87)	1.46 (0.92-2.33)		0.89 (0.68-1.16)
4th quartile (wPRS 88-100)	1.18 (0.77-1.80)		1.10 (0.88-1.38)
Model results after excluding deaths within 2 days	n = 61,631 (n = 957 of 1,316, [72.7%] deaths excluded)		n = 478,888 (n = 4,445 of 6,635 [67.0%] deaths excluded)
ED Pediatric Readiness Score:			
1st quartile (wPRS 0-58)	referent		referent
2nd quartile (wPRS 59-72)	1.24 (0.65-2.38)		1.04 (0.72-1.50)
3rd quartile (wPRS 73-87)	1.79 (1.00-3.23)		0.90 (0.67-1.20)
4th quartile (wPRS 88-100)	1.54 (0.91-2.61)		1.15 (0.88-1.48)

*wPRS = weighted Pediatric Readiness Score.

© 2023 Newgard CD et al. *JAMA Network Open.*
