# Supplementary material for: Emergency Department Pediatric Readiness and Short-term and Long-term Mortality Among Children Receiving Emergency Care
Source: JAMA Netw Open. 2023 Jan 13;6(1):e2250941. doi: 10.1001/jamanetworkopen.2022.50941 (PMC9857584; doi:10.1001/jamanetworkopen.2022.50941)
Supplement: Supplement 2. — Pediatric Readiness Study Group [file jamanetwopen-e2250941-s002.pdf]

Supplemental Online Content: Nonauthor Collaborators
*First name, last name, and suffix (if applicable) are required and will appear in PubMed.

*Group Name(s): Pediatric Readiness Study Group							
***First Name and Middle Initial(s)**	***Last Name**	***Suffix (eg, Jr, III)**	Academic Degrees	Institution	Location (city, state/province, country)	Role or Contribution, eg, chair, principal investigator	Group (if more than 1 Group listed in the byline) and/or Subgroup (eg, Steering Committee)
Ryan	Mutter		PhD	Congressional Budget Office	Washington, DC	Advisory Council	
Charles	DiMaggio		PhD	New York University	New York, New York	Collaborator	
Stephen	Wall		MD, MS	New York University	New York, New York	Collaborator	
James	Miner		MD	Hennepin Medical Center	Minneapolis, MN	Collaborator	
Brooke	Lerner		PhD	University at Buffalo	Buffalo, NY	Collaborator	
Linda	Papa		MD	Orlando Health	Orlando, FL	Collaborator	
Mark	Zonfrillo		MD, MS	Brown University	Providence, RI	Collaborator	
Nick	Mohr		MD, MS	University of Iowa Health Care	Iowa City, IA	Collaborator	
Ran	Wei		PhD	University of California, Riverside	Riverside, CA	Collaborator	
