# Supplementary material for: Emergency Department Pediatric Readiness and Short-term and Long-term Mortality Among Children Receiving Emergency Care
Source: JAMA Netw Open. 2023 Jan 13;6(1):e2250941. doi: 10.1001/jamanetworkopen.2022.50941 (PMC9857584; doi:10.1001/jamanetworkopen.2022.50941)
Supplement: Supplement 3. — Data Sharing Statement [file jamanetwopen-e2250941-s003.pdf]

Data Sharing Statement

Newgard. Emergency Department Pediatric Readiness and Short-term and Long-term Mortality Among Children Receiving Emergency Care. *JAMA Netw Open*. Published January 13, 2023. doi:10.1001/jamanetworkopen.2022.50941

Data
Data available: No

Additional Information
Explanation for why data not available: The data use agreements with state agencies required for this project do not allow data to be released to others.
